# A Standardized Porcine Model for Partial-Thickness Wound Healing Studies: Design, Characterization, Model Validation, and Histological Insights

**DOI:** 10.3390/ijms25147658

**Published:** 2024-07-12

**Authors:** Alexandru-Cristian Tuca, Ives Bernardelli de Mattos, Martin Funk, Danijel Markovic, Raimund Winter, Thomas Lemarchand, Daniela Kniepeiss, Stephan Spendel, Bernd Hartmann, Christian Ottoman, Lars-Peter Kamolz

**Affiliations:** 1 Division of Plastic, Aesthetic and Reconstructive Surgery, Department of Surgery, Medical University of Graz, 8036 Graz, Austria; alexandru.tuca@medunigraz.at (A.-C.T.); r.winter@medunigraz.at (R.W.); stephan.spendel@medunigraz.at (S.S.); lars.kamolz@medunigraz.at (L.-P.K.); 2 Department of Tissue Engineering & Regenerative Medicine (TERM), University Hospital Würzburg, 97070 Würzburg, Germany; 3 EVOMEDIS GmbH, 8036 Graz, Austria; martin.funk@evomedis.com; 4 Core Facility Experimental Biomodels, Medical University of Graz, 8010 Graz, Austria; danijel.markovic@medunigraz.at; 5 TPL Path Labs GmbH, 79111 Freiburg, Germany; tlemarchand@stagebio.com; 6 Division of General, Visceral, and Transplant Surgery, Department of Surgery, Medical University of Graz, 8010 Graz, Austria; daniela.kniepeiss@medunigraz.at; 7 BG Klinikum Unfallkrankenhaus Berlin, 12683 Berlin, Germany; bernd.hartmann@ukb.de (B.H.); otto1701de@yahoo.de (C.O.); 8 Joanneum Research Forschungsgesellschaft mbH, COREMED, 8010 Graz, Austria

**Keywords:** wound healing, animal models, histology, porcine model

## Abstract

Wound healing is a complex process that is still not fully understood despite extensive research. To address this, we aimed to design and characterize a standardized porcine model for the evaluation of wound healing, dressings, cell therapies, and pharmaceutical agents. Using a standardized approach, we examined the wound healing process in 1.2 mm-deep dermatome wounds at defined positions in 11 female pigs. Unlike previous studies that have only described/analyzed selected punch biopsies, we performed and described histological analyses along the complete wound length using quantitative morphometric methods. All animals remained fully healthy following surgery and showed no signs of infection. Our histopathological evaluation using a predetermined grading score and quantitative manual morphometry demonstrated the impact of different tissue sampling methods, sampling sites, and residual dermis thickness on wound healing. Our study presents a reproducible model for wound healing evaluation and demonstrates the usefulness of porcine models for assessing dermal and epidermal wound healing. The use of histological analyses over the complete wound length provides advantages over previous studies, leading to the possibility of a deeper understanding of the wound healing process. This model could potentially facilitate future research on novel wound dressings and local wound healing therapies.

## 1. Introduction

The process of wound healing is complex and begins immediately after an injury or tissue damage occurs. It consists of several overlapping phases, which are generally known and well described in the current literature [1]. Despite the many studies that have been conducted to date, many unresolved questions remain. Thus, further investigation of wound healing is crucial, both in vivo and in vitro. Animal experiments are necessary to address research questions and develop wound therapies, since it is challenging to recruit enough patients with comparable wounds to show statistical significance. Thus, using only clinical research, it would be impossible to gain new insights within an acceptable time frame [2].

Small mammals, including rabbits, guinea pigs, rats, and mice, are often used in wound healing studies, as they are relatively inexpensive and easy to handle [3]. However, small mammals differ from humans in a number of ways, including anatomy and physiology [4]. Regarding wound healing studies, one main difference between small mammals and humans is the thickness of their skin, especially the epidermis of fur-bearing animals, which is only 1–3 cell layers thick. Small mammals have a relatively thin epidermis and dermis, which means that their wounds heal more quickly than in humans. Another important difference is that wounds in these small mammals heal more through wound contraction than re-epithelialization [5,6,7,8].

In contrast to small mammals, there are a number of significant similarities between humans and pigs, including in terms of the anatomy and physiology of their skin [9]. In fact, Sullivan et al. (2001) showed a concordance of 78% between human wound healing studies and swine studies versus 53% between humans and small mammal studies. For instance, human and swine wound healing processes have similar wound closure mechanisms; they share the same number of healing phases, and both use re-epithelialization as the main strategy to close partial-thickness wounds [6,10]. The domestic pig also has a skin surface area similar to humans when compared to their body size. Additionally, both humans and domestic pigs have reduced hair density, comparable skin thickness [5], and similar biochemical dermal collagen [11]. Furthermore, they have similar vascularization of the hair follicles. Of course, there are also differences that should be considered; for instance, the absence of cutaneous eccrine glands in the common integument, or poor vascularization of the cutaneous glands and parts of the subepidermal plexus. Furthermore, domestic pigs present reduced variation in their skin phototype, whereas humans have a wide variety of skin tones; recently, many works have reported differences in wound healing outcomes for Fitzpatrick skin types IV–VI, especially for scarring and chronic wounds [12,13]. Despite these contrasts, a wound model using domestic pigs is still reliable and showed better concordance with human studies [14,15,16], especially regarding translational relevance [6].

There are four main categories of wound models in pigs [7,17]. These include incisional wounds, partial-thickness excisional wounds, full-thickness excisional wounds, and burn wounds. For research purposes, depending on the object to be examined and the research question, one must select the most suitable. Partial-thickness excisions, a widely studied wound type, remove the epidermis and different amounts of dermis, often leaving the bases of the sebaceous glands and hair follicles intact. These excisions are frequently made with a dermatome. They closely mimic partial-thickness burns after debridement or donor sites created from split-thickness skin grafting [14]. However, one of the limitations of wound healing experiments in pigs to date is the lack of standardization [18]. There has been no agreed protocol for these experiments so far, which makes it difficult to compare results across studies. Together, the reduced standardization and the challenges of comparing wound regeneration results call for an increased number of animals to achieve more reliable outcomes, although this raises ethical concerns, especially considering the importance of abiding by the 3R principles [19]. Since the study of wound healing requires holistic analyses that include both local and systemic responses, the use of animal models remains a necessity [14,17,20,21]. However, several factors should be considered during the experimental design in order to achieve a higher level of standardization, including animal characteristics (e.g., age and weight) and the wounds created (e.g., size, depth, and positioning). Additionally, qualitative and quantitative histological examinations set the basis for a solid understanding of the wound healing process and how different treatments can influence it. Therefore, in order to achieve a highly standardized model, it is mandatory to understand in depth the results of histomorphology analysis and how different methods can influence it.

The work reported in this study presents a thorough analysis of the histomorphology characteristics of a porcine excision wound model. For this reason, different parameters were considered, such as sampling preparation and position, as well as the reproducibility and standardization of the wounds and how different morphological aspects dictate the healing process.

## 2. Results

### 2.1. Animals and Wounds

A total of 11 female domestic pigs were included in the study. The animals were between three and five months old. The starting weight was 24–57 kg, with an average of 38.2 kg. After surgery, no animal experienced discomfort, severe pain, or adverse side effects. There was no evidence of infection or marked inflammation. None of the animals died or had to be euthanized for ethical reasons due to clinical complications. Protective bandages were applied to avoid any complications with regard to the wound dressing (e.g., detachment or dislocation) during the 7-day observation period.

### 2.2. Histological Assessment

#### 2.2.1. General

**Figure 1:**
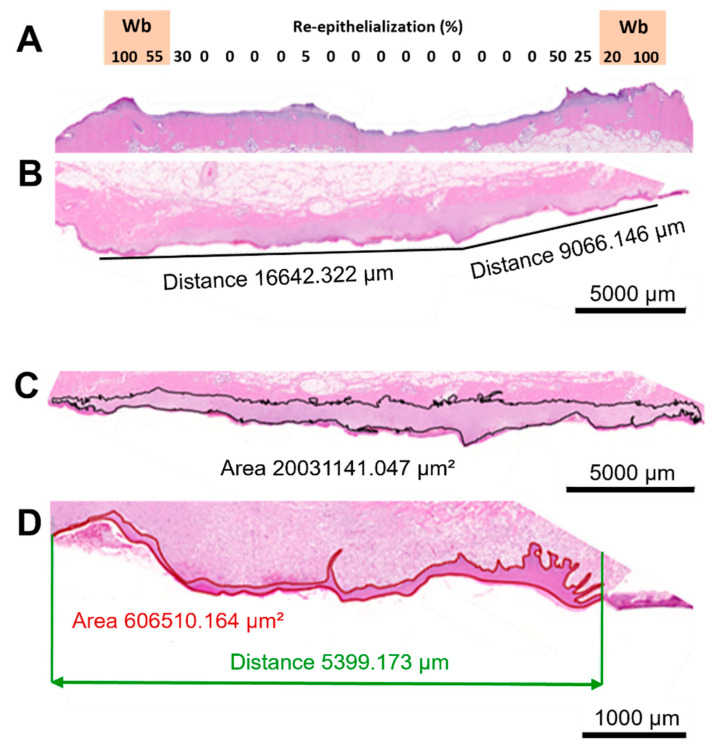
Histological cuts. (**A**) Schematic representation of how the subjective analysis was performed in the area of the wound border (Wb). (**B**) Hematoxylin and eosin (H&E) staining of the epithelial tissue showing how the wound length was estimated. (**C**) Example of H&E staining showing how the new dermal tissue area was manually outlined and estimated. (**D**) H&E staining of a tissue section showing the new epidermis area and the approximative distance.

A histopathologist used the scoring approach we have employed in former studies and used predetermined scoring criteria to undertake a blinded analysis of the H&E-stained wound sections, assessing new epidermis and new dermis [22]. To evaluate as precisely as possible the extent of new epidermis (i.e., “% re-epithelialization”), a total of 16–21 adjacent 100× microscopic fields, depending on the width of the wound, were examined using a light microscope covering the entire wound area, and the percentage of new epidermis per microscopic field was assigned to each 5% increment, creating a “new epidermis heatmap” from 0 to 100%. A global re-epithelialization percentage per histological section was calculated for each wound by averaging each microscopic field (Figure 1A). 

Quantitative manual morphometry was used to determine the wound width (wound length per histological section) and areas of newly formed tissues. The edges of the wound were defined by the thinnest detectable presence of new dermis (granulation tissue). On the wound’s surface, one straight line was drawn, or two or more were drawn and linked, to determine the approximate wound width (Figure 1B). By physically outlining the newly healed tissues, new dermal (Figure 1C) and new epidermal (Figure 1D) areas were created. By dividing the area by the measured wound width, the standardized thickness of the new epidermis and dermis were obtained.

#### 2.2.2. Dermatome Samples

**Figure 2:**
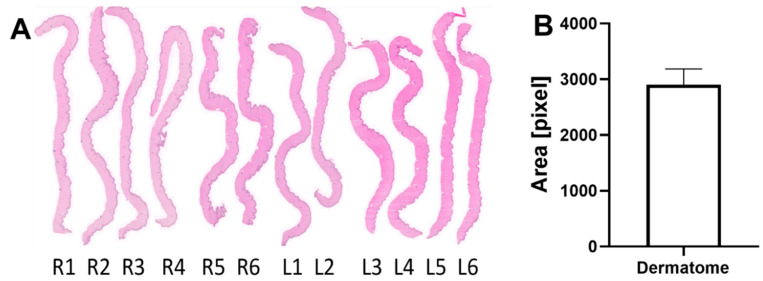
Dermatome samples. (**A**) Hematoxylin and eosin staining of representative cross-sections of dermatome samples generated from each wound. (**B**) Bar diagram of the dermatome area in pixels obtained from the histological samples (results presented as mean and SD).

The skin samples taken by dermatome showed consistent and comparable thickness and morphology (Figure 2A). The mean cross-sectional area of all dermatome skin samples was 2905 pixels with a SD of ±278.6 pixels (Figure 2B). This standardized deviation was calculated to be 9.6% of the average dermatome cross-sectional area.

#### 2.2.3. Sampling Procedure Effects and Wound Sample Position

**Figure 3:**
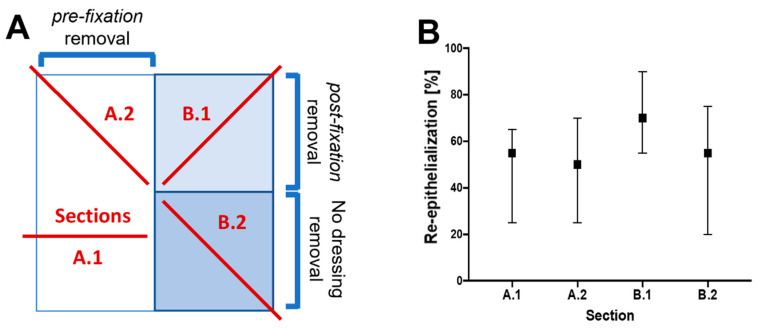
Effects of dressing removal on re-epithelialization results. (**A**) Schematic illustration showing the tissue preparation process. (**B**) Percentage of re-epithelialization obtained for each section analyzed (results presented as mean and standard deviation).

Our first evaluation, which was carried out within the wound itself, examined the possible effect of removing the dressing material before fixation for histology, as there might be an effect concerning the re-epithelialization measured, most notably, the mechanical detachment of the fragile new epithelium. Therefore, we divided the wound area (marked for orientation with a biopsy punch) in two halves: in the right half, the dressing was left in place on the wound, whereas in the left half, the dressing was removed prior to fixation. The entire area was then excised, immersed in NBF, and sent for histopathology, as described above. Cuttings were taken from different locations for histological analysis (Figure 3A). No significant differences were observed in the samples examined concerning the wound healing re-epithelialization parameter (Figure 3B).

**Figure 4:**
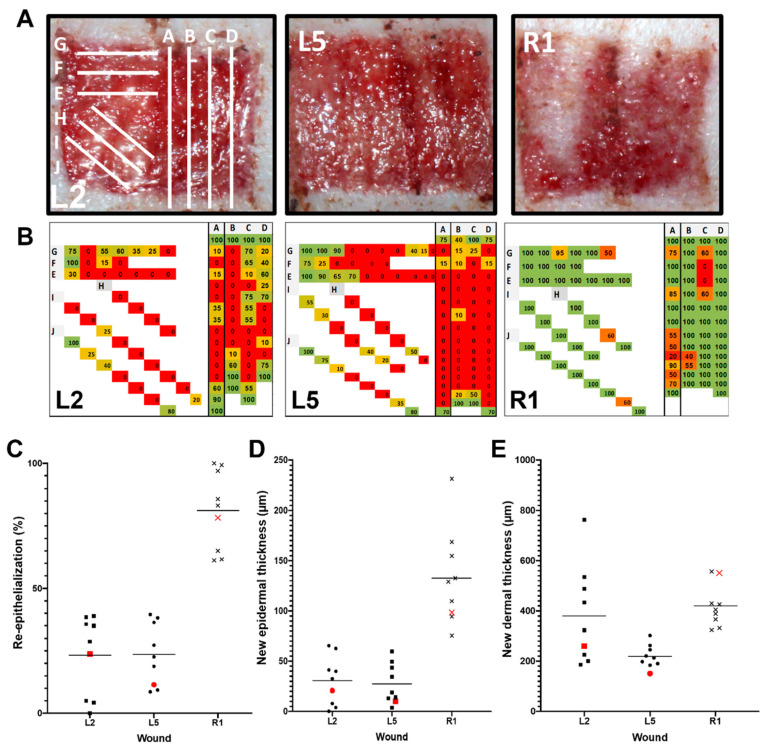
Consistency of the healing process for the same wound bed. (**A**) Macroscopic observation of three different wound sites in the same animal model using the same sampling strategy to analyze healing process variation. (**B**) Schematic illustration indicating the histopathology scoring grade provided for each histological cut. (**C**) Scatter plot showing the percentage of re-epithelialization obtained for each analyzed wound. (**D**) Scatter plot showing the standardized epithelial thickness in µm. (**E**) Scatter plot showing the standardized new dermal thickness in µm. In graphs (**C**–**E**), the lines indicate the mean value, and the red circles, squares, and crosses indicate the results obtained for sample A at the wound center.

Further, to explore possible topographical differences in wound healing within the wound, several sagittal and parasagittal or oblique cut positions were selected within the 3 × 3 cm square wound beds for histopathology. Furthermore, we wanted to evaluate whether a cutting position, such as position A (the center of the wound) in Figure 4A, is representative of the entire wound. According to the histopathology scoring analysis, the healing parameter for re-epithelialization was quite consistent, with lower re-epithelialization grades in the middle of the wound bed and expectedly high scores in the sections closest to the edge of the wound (Figure 1A and Figure 4B). Quantitative manual morphometry evaluations supported the findings by demonstrating a consistent re-epithelialization process (Figure 4C). In order to determine how this selected cutting location compared to the mean value, the results from sampling position A were highlighted with a red dot in the scatter plot figures. This alternative sampling method was also used to examine other healing parameters. Despite the presence of some outliers, the morphological characterization of the thickness of the new epidermis (Figure 4D) and the thickness of the new dermis/granulation tissue (Figure 4E) exhibited similarly consistent results.

#### 2.2.4. Body Mass Influence on the Dermal Tissue and Residual Dermal Tissue

Manual measurements were performed to assess the thickness of the unwounded dermis and the reminiscent dermal tissue under the wound bed. Intact dermal tissues on each side of the wound bed were measured in triplicate. The measurements start at the basal lamina and extend to the first cells of the hypodermis. To quantify the thickness of the residual dermis, a triplicate measurement was performed, starting at the interface between the more basophilic new dermal tissue and the residual dermis extending to the beginning of the hypodermis. The measurements obtained for the intact and residual dermis differed significantly. The difference between the two measurements was then compared to the thickness of the dermis present on the excised dermatome samples, with no apparent statistical significance. This result indicates that the missing dermis on the wound bed has roughly the same thickness as the dermis present in the excised dermatome samples. To understand how the body mass of the analyzed animals correlates to these two dermal characteristics, Pearson’s correlation was calculated for the body weight vs. the tissue thickness for the residual dermis and for the intact dermis. A very strong positive relationship was found for the comparison to the reminiscent dermis (*r* = 0.90; *p* < 0.0001), and a strong positive relationship was observed for the intact dermis (*r* = 0.82; *p* = 0.001).

#### 2.2.5. Wound Position Effects

**Figure 5:**
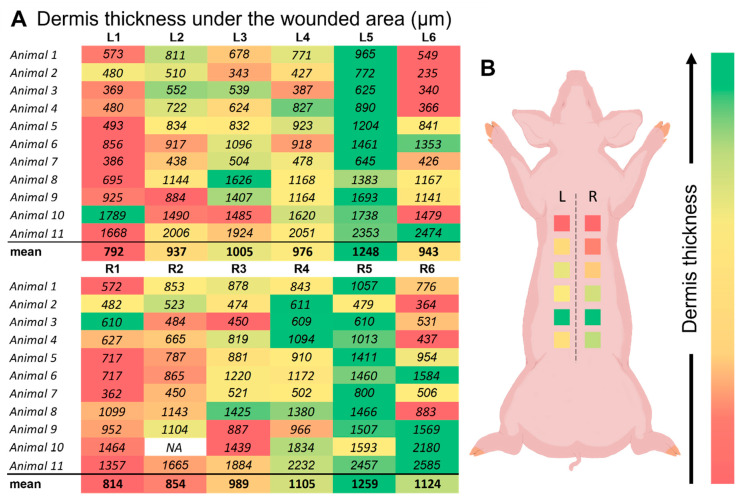
Map indicating the thickness of the dermal tissue measured under the unwounded area (NA = not applicable). (**A**) This table shows the mean value for the measured dermal tissue thickness under the unwound epidermis in µm for each position on the dorsal area (left and right sides) of the 11 animals tested The scheme indicates the position where the wounds were applied. (**B**) The schematic shows that the color system varies from red (lowest) to green (highest), with yellow in between (intermediate), indicating the relative thickness of the dermis for each position on the dorsal area of the animal.

A map displaying the mean residual thickness of the dermis under the wound for each wound site position was created using the results obtained for all 11 animals (Figure 5A). For each flank of each animal, a heat map was established using a color gradient, with the lowest values denoted by red, the middle values by orange, the high values by light green, and the highest values by dark green. A trend for the dermis thickness is apparent in this “heat map” created from the results obtained. While the dermis tissue is evidently thicker closer to the caudal section of the animal, the residual dermis thickness appears to be thinner at the regions closer to the cranium of the animal. Opposing wounds exhibited similar dermis thicknesses. As a final step, the mean value for each donor site position was determined and ranked using the same color scheme. To provide perspective on how the dermis thickness in this position is comparable to those at the other wound sites, the corresponding color scheme was applied on an illustration (Figure 5B).

#### 2.2.6. Influence of the Residual Dermis under the Wound on Healing Parameters

**Figure 6:**
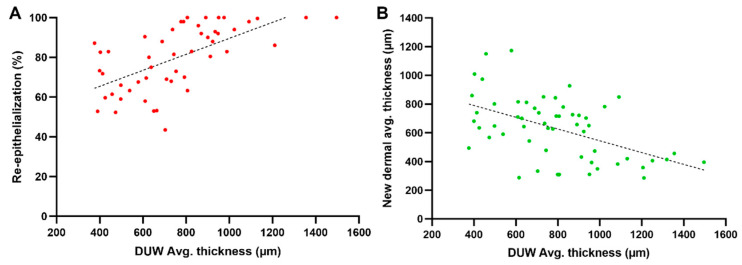
Correlation the dermal thickness under wound bed. (**A**) Graph indicating the correlation between residual dermis thickness and the percentage of re-epithelialization for each analyzed histological cut (DUW = dermis under the wound). (**B**) Graph showing the correlation between residual dermis thickness and new dermal thickness for each analyzed histological cut. Simple linear regression was calculated and plotted as a guide to help observe the tendencies of the scatter plots, with no statistical importance discovered.

Pearson’s correlation tests were conducted to determine whether the variance in residual dermis thickness had an impact on the wound donor site healing process. Thus, 56 measurements of wounds created at different dorsal positions were analyzed after seven days of treatment. The residual dermis thickness and the re-epithelialization process showed a significant and strong positive correlation (*r* = 0.63; *p* < 0.0001; *n* = 50) (Figure 6A), whereas for the regeneration of new dermal tissue, a significant and strong negative correlation was found when compared to the thickness of the residual dermis (*r* = −0.51; *p* < 0.0001; *n* = 56) (Figure 6B).

## 3. Discussion

### 3.1. Animal Selection and Wound Setting

In vivo wound models remain the most reliable models for studying the intricate processes involved in skin wound regeneration [23]. There are several reasons why pig models are particularly well suited for studying wound healing. First, pig skin is structurally very similar to human skin. This similarity allows pigs to be used to study the mechanisms of wound healing, particularly in the areas of immune response and inflammation, vascular contributions, cell proliferation and migration, and extracellular matrix remodeling. Second, pigs have a similar immune system to humans, which means that their responses to infection and inflammation are also similar [24,25]. This makes pigs ideal for studying the role of the immune system in wound healing. Finally, pigs are large animals, making them easier to handle than mice or rats. This facilitates performing surgical procedures and monitoring the healing process over time.

Based on the data collected, it can be concluded that the surgery and postoperative phases were well tolerated by the animals. There were no severe side effects, signs of pain, or other intolerance. The minimal influence of the protective bandages on the applied wound dressings suggests that the bandages were effective in protecting the wounds.

### 3.2. Histological Assessment

The use of a scoring scale introduces potential imprecision in the assessment of dermis and epidermis healing, enabling only broad, significant differences to be observed and necessitating intra-observer and inter-observer validations. The use of quantitative manual morphometry provides a measure of healing for each tissue and tissue sub-compartment, enabling comparisons between different treatments with low magnitude effects, various time points in longitudinal studies, different studies with the same design, etc. The pathologist’s evaluation of the percentage of epithelialization was highly predictive of the corresponding variable measured by interval quantitative morphometry on the epidermis.

### 3.3. Dermatome Samples

The standard deviation of the excised cross-sectional skin areas was calculated to be 9.6% of the average dermatome cross-sectional area, confirming the reproducibility and performance of the wound-making procedure with a battery-assisted dermatome. Hence, the data from the dermatome samples suggest that using a dermatome for wound setting is a consistent and reliable way of taking skin samples and setting relatively standard wounds. This dataset demonstrates that the dermatome is capable of setting wounds of consistent size, morphology, and depth (Figure 2).

### 3.4. Sampling and Histology within the Wound

The results of the intra-wound sectioning positions seem to be quite consistent, with lower re-epithelialization scores in the middle regions/center of the wound bed and expectedly high scores in the sections closest to the edge of the wound. According to the morphological evaluations, this represents a consistent re-epithelialization process. However, it should be noted that there are some outliers present. The cross-sectioning located in the middle of the donor site seems to be representative of the entire wound. However, it is possible that preparing two or three step sections could increase the representativeness of the sampling.

Furthermore, no significant differences were observed in the samples concerning the re-epithelialization healing parameter, regardless of whether the dressing material was removed before or after NBF fixation for histology. This suggests that removing the dressing material before fixation for histology can be done if needed and does not induce any significant mechanical artifacts, even in very immature epidermis. However, if it is necessary to remove it beforehand, one should do so very carefully, as this could unnecessarily injure the fragile epidermis and, as a result, significantly alter the epidermal data.

### 3.5. Body Mass Influence on the Dermal Tissue and Residual Dermal Tissue

In the present study, we investigated the thickness of the dermis in relation to the body mass of the animals. Our results showed that the thickness of the dermis positively correlated with the body mass of the animals. Moreover, integrating these results with those obtained for the relationship of the residual dermis thickness on the new dermis to new epidermis parameters, notably re-epithelialization suggests that the body mass of the animals may play an indirect role in the regeneration of the epidermis after wounding due to thicker residual dermis leading to faster subsequent re-epithelialization. Further studies are needed to confirm this finding and to elucidate the underlying mechanisms.

Consequently, if possible, to avoid any confounding factors, it is highly recommended to use age-matched and, more importantly, weight-matched individual pigs in experiments comparing several treatment modalities.

### 3.6. Position Effects

The mean residual thickness of the dermis under the wounds at different positions on the animal varies along the back of the pig. The heat map (see Figure 5) shows a trend for dermis thickness, with thicker dermis toward the caudal section and thinner dermis toward the cranial section. This provides perspective on how the dermis thickness at each position compares to the other positions. This finding is crucial in comparing wound dressings. It suggests that wounds should be compared in opposite lateral areas rather than in very different cranial and caudal areas along the back of the pig, since the dermis is thinner in the cranial area than in the caudal region.

### 3.7. Influence of the Residual Dermis under the Wound on Healing Parameters

Commonly, the rate of wound closure (% of re-epithelialization) is the major healing parameter explored in publications aiming to analyze the skin regeneration process [23,26]. However, in order to improve the standardization of the model and to better understand how the remaining tissue influences other healing parameters, we decided to include an analysis of the role of the residual tissue in skin regeneration in this study. The results of the Pearson’s correlation test evaluating the underlying dermis and the advent of new epithelium development showed a significant and strong positive correlation between residual dermis thickness and the re-epithelialization process. This indicates that wounds with a thicker residual dermis underneath the wounded area tend to achieve faster wound closure.

In examining the correlation between the thickness of the residual dermis and the thickness of the newly formed dermis, once again, a significant and strong correlation was obtained; however, this time, the correlation showed a negative effect. This suggests that the regeneration of the new dermis is based on the thickness of the residual dermis in a negative proportional relationship. It is noteworthy that no qualitative substantial difference in vascular density was observed upon histopathological evaluation. However, since vascularization density was not quantitatively examined in this study, subtle variations may not have been detected.

This phase of the study suggests that the thickness of the residual dermis is an important factor in wound healing. In the same way that the thickness of the damaged tissue has a direct impact on the healing outcome [27,28], the thickness of the dermal tissue under the wound bed influences the regeneration process. A thicker remaining dermis provides a more suitable environment for keratinocyte proliferation, offering better healing support [29,30,31]. Thus, wounds with thicker remaining dermis tend to close faster (i.e., have higher re-epithelialization rates) than wounds presenting thinner ones. For the regeneration of the dermal tissue, on the other hand, the opposite was observed, where the thickness of the residual dermis negatively impacted dermal regeneration. This negative influence was rather expected but important, since a positive influence would create hyper-granulation, a less desirable healing outcome.

### 3.8. Comparable Studies

Partial-thickness excisional wounds in pigs performed with a dermatome have been the subject of several prior studies. Table 1 outlines the important elements, including wound size, wound number, pig strain, and wound depth, to show how our study differs from these earlier studies. We may better comprehend the distinctive contributions and constraints of our study, as well as how it fits into the overall body of knowledge on partial-thickness wounds in pigs and their standardization, by contrasting and comparing these elements. Furthermore, none of these studies have performed a comparable complete histological analysis. The histological analysis performed in our study provides a comprehensive examination of the healing process and tissue regeneration following partial-thickness excisional wounds.

### 3.9. Limitations

One limitation of this study might be the young age of the pigs and their faster wound healing compared to older pigs. Additionally, in our experiments, only female animals were considered; therefore, potential variability in the healing outcomes for gilts and barrows could not be observed. Moreover, as with all animal experiments, it is also possible that the results of this study may not be fully applicable to humans, as there are differences between pigs and humans. Another limitation might be that the study was limited to a single type of wound, and it is not clear whether the results would be the same for other types of wounds. Our experiment studying the influence of the residual dermis was based on six sites that received the exact same treatment on a single animal.

## 4. Materials and Methods

### 4.1. Animal Wound Model Development

**Figure 7:**
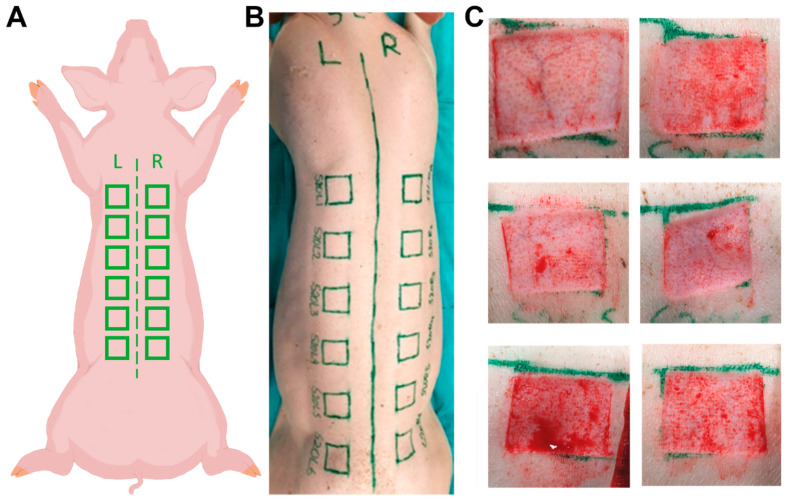
Alignment and wound setting. (**A**) Schematic dorsal position of the wounds. (**B**) Dorsal position of the wounds in vivo. (**C**) Macroscopic observation of six representative wound beds showing successful excision of epidermis and presence of a residual dermis.

The experiments were approved by the Animal Care and Use Committee (Veterinary University of Vienna, Austrian Ministry for Science and Research). For this model, we used female pigs (domestic pig—*Sus domesticus*) aged 3–5 months and weighing approximately 24–57 kg. Anesthesia and analgesia were performed on the animals by a trained veterinarian. The animal received oxygen via facemask and anesthesia with 1–2% sevoflurane (SEVOrane^®^, AbbVie GmbH, Vienna, Austria). Furthermore, via an intravenous line placed in the ear, analgesics and, if needed, additional anesthesia were administered. After anesthesia and pain management were completed, the dorsum of the animal was cleaned, and the hair was clipped. Then, the planned positioning of the wounds was marked. In total, there were 12 wounds, 6 on each side of the vertebral column. The midline (spine) and cranial height (caudal angle of the scapula) were marked where the wounds would begin. The distance to the spine or midline was 3 cm laterally. The distance between the wounds was 4 cm to ensure that they did not influence each other (Figure 7A,B). After disinfection with Octenisept^®^ (Schülke & Mayr GmbH, Norderstedt, Germany), the 12 dermatome wounds, each 3 cm × 3 cm with a depth of 1.2 mm, were set on the dorsum lateral to the spine of the pigs using a battery-operated dermatome (Figure 7C). The upper skin excised with the dermatome was stored as a sample for further investigation of its thickness in neutral buffered formalin (NBF) in biopsy cassettes. In order to stop initial bleeding from the dermatome wounds, compression was applied. After sufficient hemostasis was achieved, the study dressings (epicite^hydro^, QRSkin GmbH, Wuerzburg, Germany) were applied to the wounds and fixed in place with staples to prevent dislocation. A protective dressing was then applied and fixed to the area with staples. For analgesia, a transdermal opioid pain patch (Fentanyl 50 µg/h, Gebro Pharma GmbH, Fieberbrunn, Austria) was applied to the animal’s gluteal region. Finally, a protective bodysuit was put on to protect the bandages and wounds from alterations. No neck collarette was used. After the surgical procedure was completed, the animals were placed into their pen for recovery. For the next seven days, the animals were monitored for any signs of pain, discomfort, or distress, and if needed, treated accordingly. Seven days after sustaining the injuries, the animals were anesthetized and euthanized using a 1 mol/L dose of potassium chloride (1 M-Kaliumchlorid-Lösung, Fresenius Kabi AG, Bad Homburg vor der Höhe, Germany). Once the veterinarian confirmed the animals’ deaths with certainty, the protective body suits and protective bandages and staples were removed. The study doctor then carried out a macroscopic evaluation of the wounds, paying particular attention to infection or other abnormalities, followed by exact photographic documentation of the wounds and wound dressings. The wound areas were marked for orientation with a biopsy punch for mapping studies of the wounds. On some wounds, the wound dressings were partially removed for evaluation of the influence of manipulation of the wound before histological assessment. The wounds were then excised epifascially in toto for further histological investigation by a veterinary pathologist. The skin samples, including the samples of microtome-excised superficial skin taken on day 1, were sent to the pathology laboratory (TPL Path Labs GmbH, now StageBio, Freiburg, Germany).

### 4.2. Histological Analysis

#### 4.2.1. General

Tissues were fixed with NBF for a maximum of 72 h and routinely trimmed according to the standard protocol, embedded in paraffin, cut at a nominal thickness of 3 µm, and stained with hematoxylin and eosin (H&E) using standard operating procedures. A histopathologist then conducted an analysis of the slides, blinded to the treatment modalities. He concentrated the analysis on the width of each wound and the process of repair of primarily two tissues: the epidermal tissue (analyzing the epidermal area, the percentage of re-epithelialization, and the standard thickness of epidermis) and the dermal tissue (analyzing the new granulation tissue area and the thickness scoring for the new dermis on each harvested tissue sample). Additionally, Zen 3.3 blue edition software (Zeiss Microscopy, Jena, Germany) was used to conduct hand measurements of the morphological characteristics of the new epidermis (percent coverage and average “standard” thickness) and the average thickness of the new and preexisting dermis. The standard thicknesses of the new epidermis and the new dermis were calculated by dividing the new epidermis or new dermis/granulation tissue areas, respectively, by the measured wound length per section (wound width)—see Figure 1C,D.

#### 4.2.2. Sampling and Histology within the Wound

**Figure 8:**
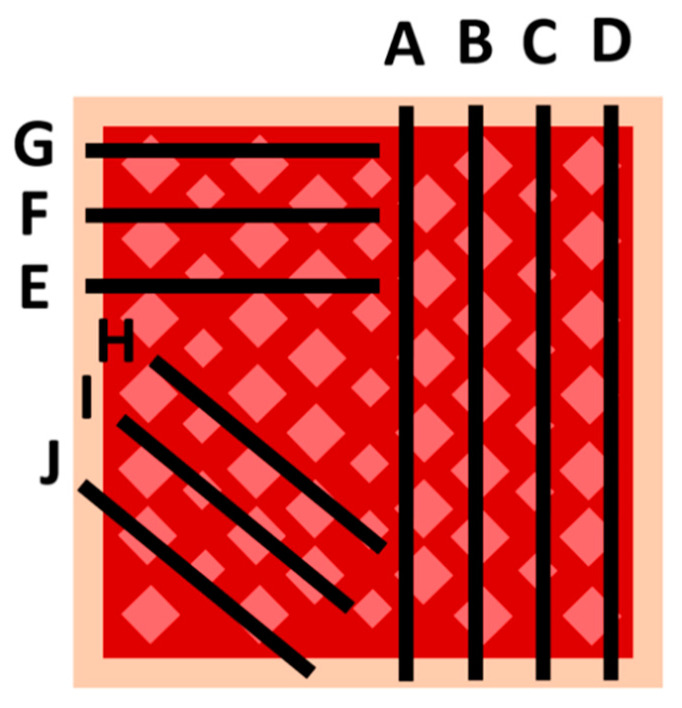
Histological cuts. Schematic sampling strategy used to analyze healing process variation.

Each wound site was sampled, including an approximately 0.5 cm-wide edge of normal preexisting skin adjacent to the wound sites. In the current study, we evaluated the dermatome-induced intra-wound healing process occurring within the wound to examine any changes in healing parameters. An overview of the wound bed at several locations was made possible by macroscopic investigation. Based on this observation, an apparently uneven process of re-epithelialization was suspected. To test this hypothesis, a sampling strategy was designed to examine the influence of within-wound positional locations on healing degree status. This was carried out by choosing several allocations of histological cuts (i.e., the map of suggested slices forwarded to the trimming laboratory before paraffin embedding) within the wound sites (Figure 8). Furthermore, a punch biopsy was conducted cranially outside the wound to permit wound site orientation by the pathologist.

#### 4.2.3. Statistical Analysis

The results of the quantitative variables of the study were expressed as the mean ± standard deviation (SD), and significant differences were calculated using Student’s *t*-test, with significance indicated by *p* < 0.05. Pearson’s correlation analysis was used to analyze the relationship between the different quantitative healing parameters. The results are expressed in terms of Pearson’s correlation coefficients and *p*-values. Data analysis was performed using GraphPad Prism 9 (GraphPad Software, La Jolla, CA, USA).

## 5. Conclusions

In conclusion, our study has contributed significantly to the field of wound healing by designing and characterizing a standardized porcine model for wound healing evaluation. We implemented a unique approach by performing histological analyses over the complete wound length, in contrast to previous studies, which have only described/analyzed selected punch biopsies. This method provided unprecedented insights into the complex process of wound healing and allowed us to identify the impact of various factors on wound healing. Another important conclusion with potential clinical relevance of this study suggests that a wound’s ability to heal is significantly influenced by the thickness of the residual dermis under the wounded area. In comparison to wounds with thinner residual dermis, wounds with thicker residual dermis typically healed more quickly (i.e., re-epithelialize more rapidly). This is probably because the thicker dermis offers a better physical and/or chemical environment for the regeneration of new epidermal tissue, perhaps better supplying the necessary growth factors through a better vascular network. This hypothesis could be tested in further studies. The clinical significance of this study lies in its introduction of a new parameter, residual dermis thickness, which has the potential to enhance wound healing by informing and directing treatment options, ultimately resulting in improved patient outcomes.

Additionally, our findings prove that the current porcine wound model is a reproducible tool for evaluating dermal and epidermal wound healing. This model could serve as a useful platform for future research on novel wound dressings and local wound healing therapies. Overall, our study adds to the current understanding of wound healing and provides a foundation for further investigation in this area. By providing insights into the underlying mechanisms of wound healing, this research may pave the way for the development of more effective therapies and treatments for wound healing in both humans and animals. Furthermore, the development of a standardized porcine model for wound healing evaluation will enable researchers to better understand the complexities of wound healing and ultimately improve outcomes for patients suffering from wounds.

## Figures and Tables

**Table 1:** Comparison of studies on porcine wound models.

Year	Author	Number of Wounds per Animal	Weight Range (kg)	Wound Area (cm)	Wound Depth (mm)	Biopsy Size
1981	Stanley et al. [32]	not specified	5–9	0.7 × 1	0.3	not specified
1992	Pirone et al. [33]	12	15–20	2.2 × 2.2	0.5	not specified
2000	Olson et al. [34]	72	20–25	1 × 2	0.4	2 × 3 cm
2003	Singer et al. [23]	~38	20–30	2.5 × 2.5	0.6	not specified
2003	Singer et al. [35]	32	20–30	2.5 × 2.5	0.3–0.9	not specified
2007	Singer et al. [36]	10	40	2.5 × 2.5	0.6	1.25 × 1.25 cm
2010	Faucher et al. [37]	2	18.14	not specified	0.56 & 0.76	not specified
2012	Peura et al. [38]	8	18–26	4 × 5	0.76	5 × 6 cm
2013	Masella et al. [39]	24	25	4 × 2	0.15	2.5 × 5.5 cm
2013	Mauskar et al. [40]	12	30–55	7.6 × 7.6	~1.5	9 times 3 mm punch
2014	Travis et al. [41]	6	30–55	7.6 × 7.6	~1.5	9 times 3 mm punch
2019	Wlaschin et al. [42]	4	28–32	2.5 × 2.5	0.5	0.8 × 5 cm
2019	Schiefer et al. [43]	3	25.8 (±2.5)	2.4 × 2.4	0.5	not specified
2020	Connolly et al. [44]	6	10–15	5 × 5	0.1	5 × 5 cm
2023	Nakano et al. [45]	4	~27.5	8 × 10	1.5	6 times 5 mm punch
Present study	Tuca et al.	12	24–57	3x3	1.2	4 × 4 cm

## Data Availability

The data presented in this study are available on request from the corresponding author. The data are not publicly available due to organizational reasons.
